# Human Neural Stem Cells Genetically Modified to Overexpress Akt1 Provide Neuroprotection and Functional Improvement in Mouse Stroke Model

**DOI:** 10.1371/journal.pone.0005586

**Published:** 2009-05-18

**Authors:** Hong J. Lee, Mi K. Kim, Hee J. Kim, Seung U. Kim

**Affiliations:** 1 Division of Neurology, Department of Medicine, UBC Hospital, University of British Columbia, Vancouver, Canada; 2 Medical Research Institute, Chungang University College of Medicine, Seoul, Korea; 3 Department of Pharmacology, Dankook University School of Medicine, Cheonan, Korea; Universidade Federal do Rio de Janeiro (UFRJ), Instituto de Biofísica da UFRJ, Brazil

## Abstract

In a previous study, we have shown that human neural stem cells (hNSCs) transplanted in brain of mouse intracerebral hemorrhage (ICH) stroke model selectively migrate to the ICH lesion and induce behavioral recovery. However, low survival rate of grafted hNSCs in the brain precludes long-term therapeutic effect. We hypothesized that hNSCs overexpressing Akt1 transplanted into the lesion site could provide long-term improved survival of hNSCs, and behavioral recovery in mouse ICH model. F3 hNSC was genetically modified with a mouse Akt1 gene using a retroviral vector. F3 hNSCs expressing Akt1 were found to be highly resistant to H_2_O_2_-induced cytotoxicity *in vitro*. Following transplantation in ICH mouse brain, F3.Akt1 hNSCs induced behavioral improvement and significantly increased cell survival (50–100% increase) at 2 and 8 weeks post-transplantation as compared to parental F3 hNSCs. Brain transplantation of hNSCs overexpressing Akt1 in ICH animals provided functional recovery, and survival and differentiation of grafted hNSCs. These results indicate that the F3.Akt1 human NSCs should be a great value as a cellular source for the cellular therapy in animal models of human neurological disorders including ICH.

## Introduction

Two major types of stroke are cerebral infarction (ischemia) and intracerebral hemorrhage (ICH). ICH causes severe neurological deficits and extensive death rate in patients. Since medical therapy against ICH such as mechanical removal of hematoma, prevention of edema formation by drugs and reduction of intracranial pressure shows only limited effectiveness, alternative approach is required such as stem cell-based cell therapy [1], [2].

Recent progress in stem cell biology has opened up a new way to therapeutic strategies to replace lost neural cells by transplantation of neural stem cells (NSCs) in CNS injury and disease [3]–[8]. Previous studies have indicated that NSCs or neural progenitor cells engrafted in animal models of stroke survive and ameliorate neurological deficits in the animals [9]–[17]. Among these studies, human neural progenitor cells isolated from fetal brain have been transplanted into the brain of stroke animal models and found to restore brain function [13], [14]. This approach, however, is not widely acceptable for stroke patients because of moral, religious and logistic problems associated with the use of human fetal tissues. In addition, primary human NSCs derived from fetal tissues can be provided for only a limited time before they undergo senescence, and it is difficult to secure sufficient numbers and homogeneous populations of human NSCs from fetal brain. These problems can be circumvented by the use of stable, permanent cell lines of human NSCs. We have previously reported that human NSC line ameliorate neurological deficits in animal models of Parkinson disease [18], Huntington disease [19], [20], amyotrophic lateral sclerosis [21] and lysosomal storage disease [22] following their transplantation into the brain or spinal cord. In stroke animal models, intravenously transplanted human NSCs migrated selectively to the damaged brain sites caused by ischemia and ICH, differentiated into neurons and astrocytes, and promoted functional recovery in these animals [9]–[12], [15]–[17]. However, low survival rate of grafted F3 NSCs in ischemia and ICH rats in the previous studies is a grave concern; less than 50% of grafted NSCs survived in ICH mice at 2-weeks post transplantation and 30% at 8-weeks [15], [16].

One possible way to promote extended survival of transplanted NSCs in animal brain is to modulate properties of the NSCs, and this might be accomplished by over-expressing Akt1 protein which is known as a general mediator of cell survival signals in the NSCs. Akt, a serine/threonine kinase, plays a critical role in the modulation of cell proliferation, growth, and survival. The PI3K-Akt signal pathway is well-known for the cell survival and it exhibits anti-apoptotic effects against a variety of apoptotic paradigms including withdrawal of extracellular signaling factors, oxidative and osmotic stress, irradiation and ischemic shock [23]–[26]. Previous studies have demonstrated that overexpression of Akt prevents cerebellar granule cells from apoptotic cell death during growth factor withdrawal [23], and promotes cell survival during free radical exposure to free radical or hypoxia in hippocampal neurons [27]–[29].

Considering evidence of functional recovery in stroke animals following brain transplantation of human NSCs and Akt1 protein as a general mediator of survival signals, the present study is designed to investigate whether human NSCs overexpressing Akt1 can lead to the prolonged cell survival of grafted human NSCs and functional recovery in the mouse ICH stroke model.

## Materials and Methods

### Cell culture

Primary dissociated cell cultures of fetal human brain tissues of 14 weeks gestation were prepared as described previously [30], [31]. The cells were grown in T25 flasks in Dulbecco's modified Eagle medium with high glucose (DMEM; HyClone, Logan, UT), 5% fetal bovine serum (FBS) and 20 µg/ml gentamicin (Sigma, St Louis, MO) (Sigma). The medium was changed twice a week. The permission to use the fetal tissues was granted by the Clinical Research Screening Committee involving Human Subjects of the University of British Columbia, and the fetal tissues were obtained from the Anatomical Pathology Department of Vancouver General Hospital.

Stable, immortal human neural stem cell line, HB1.F3 (F3), was generated from the primary fetal human brain cell culture as described previously [15], [32]–[34]. PA317 amphotropic packaging cell line was infected with the recombinant replication-incompetent retroviral vector pLNX.v-myc, and the supernatants from the packaging cells were used to infect NSCs in human telencephalon cultures. Stably transfected colonies were selected by neomycine resistance. Several stable clones of human NSCs (hNSCs) were isolated, and one of them, HB1.F3 (F3 hereafter), was expanded for the present study. F3 hNSCs express ABCG2, nestin and Musashi1, which are cell type specific markers for NSCs [15], [34].

To generate Akt1 overexpressing hNSC line (F3.Akt1), Akt1 cDNA (Upstate, Charlottesville, VA) was ligated into multiple cloning sites of the retroviral vector pLHCX (Clontech, Mountain View, CA). Before the ligation, mouse Akt1 cDNA was PCR-amplified by using forward primer 5′-CTAGTTAAGCTTATGGGGAGCAGC-3′; reverse primer 5′- GATATGATCGATTGATC AGAGGGTTTA -3′. PA317 amphotropic packaging cell line was infected with the recombinant retroviral vector, and the supernatants from the packaging cells were added to the F3 hNSCs. Stably transfected colonies were selected by hygromycin resistance.

### RT-PCR analysis

Reverse transcription was performed with M-MLV reverse transcriptase (Promega, Madison, WI) for 1 hr at 42°C, inactivated for 15 min at 95°C and cooled at 4°C. PCR reaction solution consisted of DNA polymerase buffer, containing cDNA 1 µL, 5 mM MgCl_2_, 1 mM dNTPs, 10 pM primers and 2.5 units Taq polymerase (Promega). The cDNA was amplified using 30 PCR cycles and RT-PCR products were separated electrophoretically on 1.2% agarose gel containing ethidium bromide and visualized under UV light. The primers used for the RT-PCR for nestin, neurofilament (NF)-L, NF-M, NF-H, glial fibrillary acidic protein (GFAP), GAPDH and Akt1 are listed in Table 1.

**Table 1:** PCR primer sequences for cell type-specific markers (all human).

Gene	Sequence
Akt1	Sense: 5′-ACCTCTGAGACTGACACCATG-3′
	Antisense: 5′-CACTGGCTGAGTAGGAGAAC-3′
Nestin	Sense: 5′-CTCTGACCTGTCAGAAGAAT-3′
	Antisense: 5′-GACGCTGACACTTACAGAAT-3′
NF-L	Sense: 5′-TCCTACTACACCAGCCATGT-3′
	Antisense: 5′-TCCCCAGCACCTTCAACTTT-3′
NF-M	Sense: 5′-TGGGAAATGGCTCGTCATTT-3′
	Antisense: 5′-CTTCATGGAAGCGGCCAATT-3′
NF-H	Sense: 5′-CTGGACGCTGAGCTGAGGAA-3′
	Antisense: 5′-CAGTCACTTCTTCAGTCACT-3′
GFAP	Sense: 5′-GCAGAGATGATGGAGCTCAATGACC-3′
	Antisense: 5′-GTTTCATCCTGGAGCTTCTGCCTCA-3′
GADPH	Sense: 5′-CATGACCACAGTCCATGCCATCACT-3′
	Antisense: 5′-TGAGGTCCACCACCCTGTTGCTGTA-3′

### Hydrogen peroxide treatment

F3 and F3.Akt1 cells were plated 1×10^4^ cells per well in 96 well-plates (Falcon, Becton Dickinson, Franklin lakes, NJ) with DMEM containing 5% FBS and incubated for overnight. Hydrogen peroxide (Sigma) was added to each well containing F3 and F3.Akt1 cells to give a final H_2_O_2_ concentration in the range from 0 to 0.5 mM. Control wells contained normal medium. Cells were left for 6 hr and 24 hr for viability assay and for 6 hr for western blot analysis.

### Cell viability assay

Cell viability was determined by the conversion of MTT [3-(4,5-dimethylthiaxol-2-yl)-2,5 diphenyl tetrazolium bromide] to formazan utilizing NADH and NADPH pyridine nucleotide cofactors. After stimulation with H_2_O_2_ at different dose for 6 or 24 hr, MTT [2 mg/ml in phosphate buffered saline (PBS)] was added to each well and incubated for 2 hr. The media containing MTT was removed, 100 µL of dimethyl sulfoxide (DMSO, Sigma) added to each well and absorbance read at 570 nm.

### Western blot analysis

Western blot analysis was performed with 50 µg total protein extract separated on 10% SDS-PAGE gels that were subsequently transferred to polyvinylidene difluoride (PVDF) membrane (Millipore, Billerica, MA). Blocking of membranes with 5% skim milk in TBST and washed in TBST, membranes were incubated with anti-phospho-Akt1-Thr 308 (1∶500, Upstate), anti-caspase-3 (1∶1000, Chemicon) and anti-beta-actin antibody (1∶10,000, Santa Cruz) at 4°C overnight, membrane washed in Tris-buffered saline containing 0.05% tween 20 for 1 hr at RT, then incubated in secondary antibody [horseradish peroxidase-conjugated anti-rabbit or anti-mouse IgG] (Sigma) for 2 hr at RT. Immunoreactive bands were detected by chemiluminescence using ECL system. Western blots analyses were performed on samples from twice separate experiments.

### Mouse intracerebral hemorrhage stroke model

All experimental procedures were approved by the Animal Care Committee of the University of British Columbia. ICH was induced by stereotaxic, intrastriatal administration of bacterial collagenase by previously described methods [12], [16], [17]. In brief, after an intraperitoneal injection of 1% ketamine (30 mg/kg) and xylazine hydrochloride (4 mg/kg), the mice were placed in a stereotaxic frame (Kopf Instruments, Tujunga, CA). A burr hole was made, and a 30-gauge needle was inserted through the burr hole into the striatum (0.1 mm posterior, 4.0 mm ventral, and 2.0 mm lateral to the bregma). ICH was induced by the administration of collagenase type IV (0.5 µL saline containing 0.078 U, Sigma) over a period of 5 min. After remaining in place for another 3 min, the needle was gently removed.

### Brain transplantation

F3 and F3.Akt1 hNSCs were dissociated into single cells by a brief trypsin treatment and suspended in PBS at 4×10^7^ cells/0.1 ml and kept on ice until transplanted. Randomly selected ICH mice of one week after ICH surgery received 2 µL (2×10^5^ cells) of F3.Akt1 cell suspension (n = 9), F3 cell suspension (n = 9) and killed F3 cell suspension (n = 10). F3 cells in glass tube were killed by placing the tube in boiling water for 1 min, injected slowly for 5 minutes into overlying cortex of the hemorrhage lesion (0.1 mm posterior, 2.0 mm ventral, and 2.0 mm lateral to the bregma). In another control group, 2 µL of PBS was injected into the ICH mice (n = 10). Immunosuppressant was not used in any of the animals. In order to identify the migration potential to the lesion site of grafted cells, F3.Akt1 hNSCs were infected with an adenovirus vector encoding LacZ gene (pAV.LacZ) in vitro at 100 MOI (PU/cell) for 24 hr before transplantation (n = 2).

### Behavioral Testing

Behavioral testing was performed weekly with the rotarod (Harvard Instrument) by 2 individuals blinded to mice treatment status [16], [17]. In the rotarod test, mice were placed on the rotarod cylinder, and the time the animals remained on the rotarod was recorded. The speed was slowly increased from 10 to 40 rpm within a period of 2 min. The trial was ended if the animal fell off the rungs or gripped the device and spun around for 2 consecutive revolutions. The animals were trained for 3 days before ICH operation. The maximum duration (in seconds) on the device was recorded with 3 rotarod measurements 1 day before ICH induction. Motor test data are presented as percentages of the maximal duration compared with the internal baseline control (before ICH). The modified limb-placing test is a version of a test previously described [16], [17]. The test consists of 2 limb-placing tasks that assess the sensorimotor integration of the forelimb and the hind limb by checking responses to tactile and proprioceptive stimulation. First, the mouse is suspended 10 cm over a table, and the stretch of the forelimbs toward the table is observed and evaluated: normal stretch, 0 points; flexion with a delay (2 sec) and/or incomplete, 1 point; abnormal flexion, 2 point. Next, the mouse is positioned along the edge of the table, with its forelimbs suspended over the edge and allowed to move freely. Each forelimb (forelimb, second task; hind limb, third task) is gently pulled down, and retrieval and placement are checked. Finally, the mouse is placed toward the table edge to check for lateral placement of the forelimb. The 3 tasks are scored in the following manner: normal performance, 0 points; performance with a delay (2 sec) and/or incomplete, 1 point; no performance, 2 points. A total of 9 points means maximal neurological deficit, and 0 points means normal performance. Additionally, the body weights of all animals were checked weekly for 8 weeks.

### Histology and immunohistochemistry

Histology and immunohistochemistry of brain sections were performed as described previously [16], [17]. At the end of behavioral testing, each animal was anesthetized and perfused through the heart with cold saline followed by 4% paraformaldehyde in 0.1 M phosphate buffer. The brains were post-fixed in same fixative for 24 hr, followed with followed with cryoprotection in 30% sucrose for 24 hr and then 30 µm sections were prepared on a cryostat (Leica CM 3000). The sections through the needle entry site which was identifiable on the brain surface, and sites 1.0 mm anterior and 1.0 mm posterior to plane were processed for X-gal staining to analyze the hemisphere area. These sections are representative of the core of the ICH lesion. The morphometric analyses involved computer-assisted hand delineation of the area of the striatum, cerebral cortex, and ventricles, as well as the whole hemisphere. Adjacent serial coronal sections were processed for double immunofluorescence staining of human nuclear matrix antigen (hNuMA, 1∶100, mouse monoclonal, Oncogene) and antibodies specific for cell type specific markers. Antibodies specific for neurofilament low molecular weight protein (NF-L, 1∶1000, rabbit, Chemicon), neurofilament high molecular weight protein (NF-H, 1∶1000, rabbit, Chemicon), microtubule associated protein-2 (MAP2, 1∶500, rabbit, Chemicon), glial fibrillary acidic protein (GFAP, 1∶1000, rabbit, DAKO, Carpinteria, CA), and phospho-Akt1 (1∶100, rabbit, Upstate) were used for cell type identification of neurons and astrocytes. Brain sections were incubated in mixed solution of primary antibodies overnight at 4°C as free floating sections, followed by mixed secondary antibodies of Alexa Fluor 488-conjugated anti-mouse IgG (1∶1000, Molecular Probes, Eugene, OR) and Alexa Fluor 594-conjugated anti-rabbit IgG (1∶1000, Molecular Probes) for 1 hr at RT. Negative control sections from each animal were prepared for immunohistochemical staining in an identical manner except the primary antibodies were omitted. Stained sections were then examined under an Olympus confocal laser scanning biological microscope (Olympus, Tokyo, Japan).

### Stereological cell counts

Total number of human NuMA-positive F3 (n = 3) and F3.Akt1 (n = 3) hNSCs in the brain sections from ICH animals was determined by stereological estimation as described previously [16], [17]. The sections used for cell count covered the entire striatum with hemorrhage lesion and overlying cortex. This generally yielded six or seven sections in a series. Sampling was done using the Computer assisted stereological toolbox system, version 2.1.4 (Olympus), using an Olympus BX51 microscope, a motorized microscope stage (Prior Scientific, Rockland, NY) run by an IBM compatible computer, and a microcator (Heidenhain ND 281B, Schaumberg, IL) connected to the stage and feeding the computer with the distance information in the z-axis. The counting areas were delineated at a 1.25× objective and generated counting areas of 150×150 µm. A counting frame (1612 µm^2^) was placed randomly on the first counting area and systemically moved through all counting areas until the entire delineated area was sampled. Actual counting was performed using a 100× oil objective. Guard volumes (4 µm from the top and 4–6 µm from the bottom of the section) were excluded from both surfaces to avoid the problem of lost caps, and only the profiles that came into focus within the counting volume (with a depth of 10 µm) were counted. The estimate of the total number of HuNuMA-positive F3 and F3.Akt1 calculated according to the optical fractionator's formula [35].

### Statistical analysis

The statistical significance between group comparisons for behavioral data was determined by one-way ANOVA and two-way ANOVA. P values<0.001 were considered to be statistically significant (version 12.0, SPSS, Chicago, IL).

## Results

### Stable human neural stem cell line overexpressing Akt1

**Figure 1:**
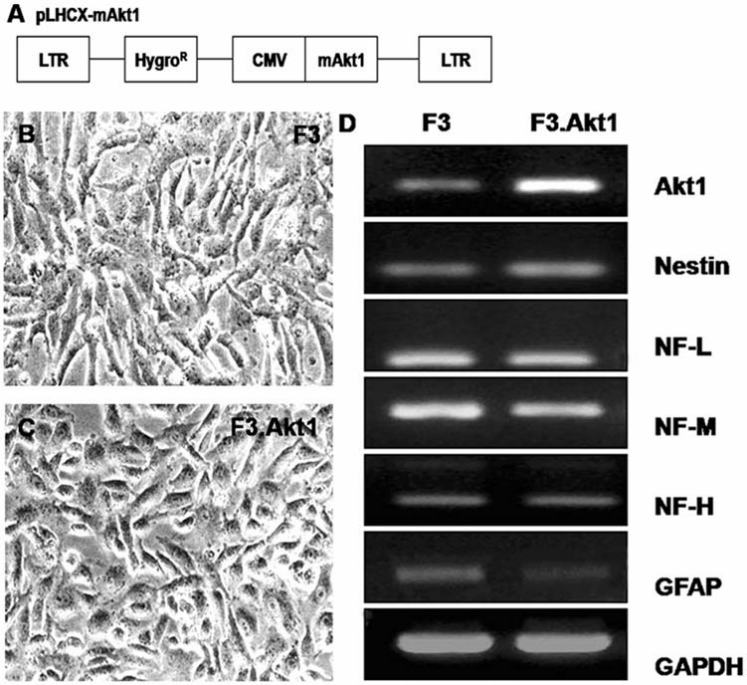
Characterization of human NSC lines. A: The retroviral vector encoding Akt1 (pLHCX.Akt1) used in the present study for the generation of HB1.F3.Akt1 (F3.Akt1) human neural stem cell (NSC) line. B and C: Phase contrast microscopy of F3 and F3.Akt1 human NSCs. Bar inciates 20 µm. D: Gene expression of cell type-specific markers as studied by RT-PCR in F3 and F3.Akt1 human NSCs. Both of F3 and F3.Akt1 NSC lines express cell type-specific markers Nestin (for neural stem cells), NF-L, NF-M and NF-H (for neurons), GFAP (for astrocytes) and Akt1.

F3 hNSC line was infected with a retroviral vector encoding mouse Akt1 gene (Figure 1A), and clones resistant to hygromycin were selected and expanded. One of the clones was chosen and used in the present study. The morphology of the selected hNSC line, F3.Akt1 does not differ from the parental F3 hNSCs with bipolar- or multipolar-morphology (Figures 1B, C). Results of RT-PCR analysis of mRNAs isolated from F3 and F3.Akt1 cells are shown in Figure 1D. Transcripts for nestin (an NSC specific marker), neurofilament triplet proteins (NF-L, NF-M and NF-H, cell type-specific markers for neurons), glial fibrillary acidic protein (GFAP, a specific marker for astrocytes) and Akt1 are all expressed by both F3 and F3.Akt1 cells. However, GFAP band at F3.Akt1 lane detected was lighter than F3 parental cells. In addition transcriptional level of Akt1 gene is higher in F3.Akt1 cells as compared to parental F3 cells.

### Hydrogen peroxide (H_2_O_2_)-Induced cell death

**Figure 2:**
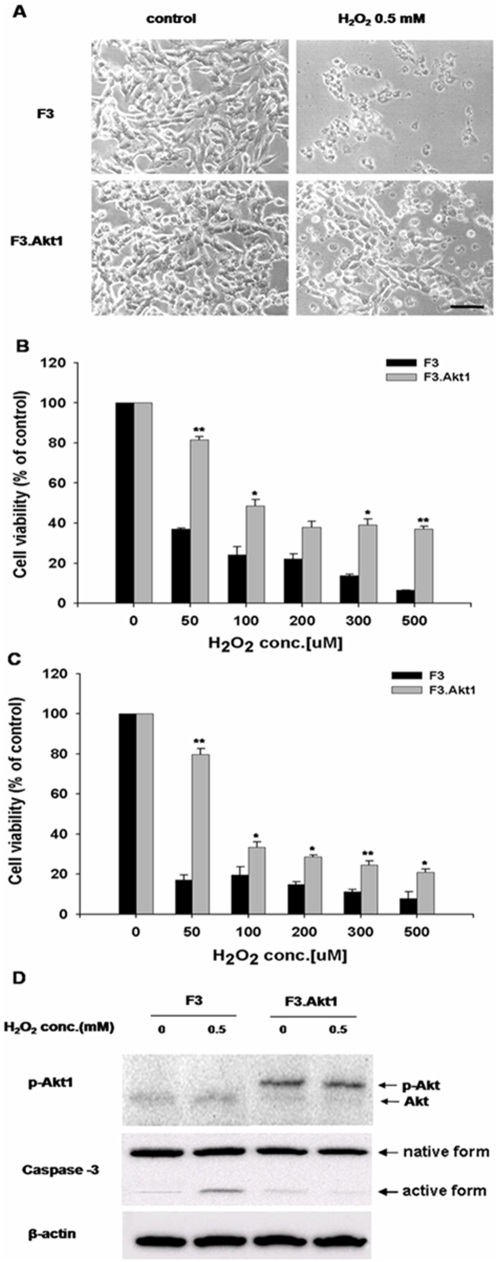
Cell viability increases to H_2_O_2_–induced oxydative stress conditions and Akt1 phosphorylation in F3.Akt1 human NSCs. A: Phase contrast microscopy of F3 and F3.Akt1 human NSCs following exposure to 0.5 mM H_2_O_2_ for 6 hr. F3.Akt1 NSCs survived well as compared to the parental F3 NSCs. B and C: F3.Akt1 NSCs were found to show resistance to H_2_O_2_-induced cell death as compared to control parental F3 NSCs at 6 hr (B) and 24 hr (C) respectively. D: Western blot analyses of protein levels of phopho-Akt1 and caspase-3 enzymes in F3 and F3.Akt1 NSCs following H_2_O_2_ treatment. F3.Akt1 NSCs showed an increased level of Akt1 phosphorylation, while the level in activation form of caspase-3 was reduced under the H_2_O_2_ treatment (* p<0.05, ** p<0.001).

To determine the protective effects of Akt1 on the H_2_O_2_-induced cell death in hNSCs, F3 and F3.Akt1 hNSCs were exposed to varying concentration of H_2_O_2_. F3.Akt1 cells showed a higher survival rate to H_2_O_2_ -induced cell death as compared to the F3 controls (Figures 2A–C). Whether H_2_O_2_ induces changes in phosphorylation status of Akt1 and caspase-3 cleavage in F3 and F3.Akt1 cells was investigated. When F3 and F3.Akt1 cells were exposed to 0.5 mM H_2_O_2_ for 6 hr, phosphorylated form of Akt1 and inactivated form of caspase-3 were found in F3.AKt1 cells (Figure 2D), while F3 cells showed the opposite expression pattern (Figure 2D). An increase in active fragment of caspase 3 (20 kDa) was found in F3 cells following H_2_O_2_ treatment. These results indicate that the enhanced cell survival of F3.Akt1 cells as compared to parental F3 cells following H_2_O_2_ exposure is due mainly from overexpression of Akt1 and its phosphorylation in F3.Akt1 cell line. And there might be some mediators from Akt1 to caspase-3 in signal pathway; we could consider that phospho-Akt1 finally inhibits caspase-3 cleavage and activation, thereby provides neuroprotection of the hNSCs.

### Functional recovery in ICH animals by hNSC transplantation

**Figure 3:**
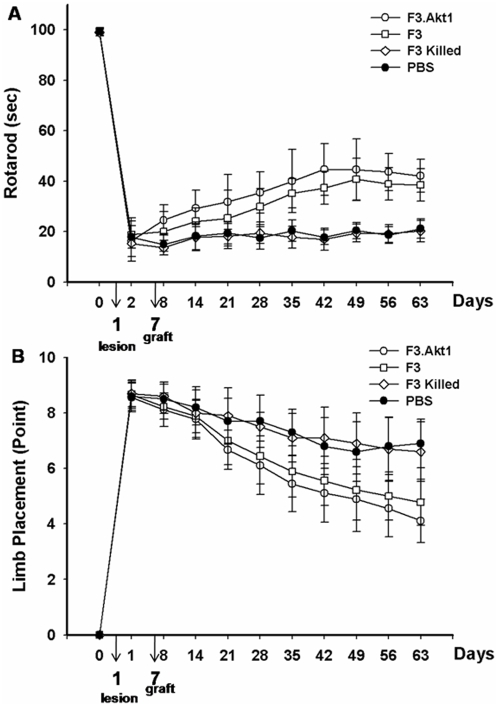
Behavioral improvement demonstrated in intracerebral hemorrhage (ICH) mice transplanted with F3 or F3.Akt1 human NSCs. A: Rotarod test. F3.Akt1-transplanted group showed better performance than PBS controls or F3 cell group, killed F3.Akt1 cell group 8 days onward, and these benefits continued up to 8 weeks post-transplantation (* P<0.05). B: In the modified limb placement test, F3.Akt1-transplanted group showed better performance than PBS, F3 or killed F3.Akt1 group (* P<0.05).

Motor performance of ICH animals receiving PBS, F3 Killed, F3 or F3 Akt1 hNSCs was determined by the rotarod and neurology score (Figures 3A, B). The ICH mice receiving F3.Akt1 hNSCs showed improved behavioral performance on the rotarod compared with the PBS, F3.killed control groups, and the effect persisted for at least up to 8 weeks post-transplantation (PT, the point at which animals were sacrificed) (Figure 3A). Significant difference in rotarod test performance in F3.Akt1 vs F3 groups detected during the period of only 5 weeks PT (P<0.05). The F3.Akt1 transplantation group also showed marked improvement in the limb placement beginning 2 weeks PT and persisting for at least up to 8 weeks (Figure 3B). No significant difference in behavioral performance in F3.Akt1 vs F3 groups detected during the period of 1∼8weeks PT.

### Transplanted NSCs differentiate into neurons and astrocytes

**Figure 4:**
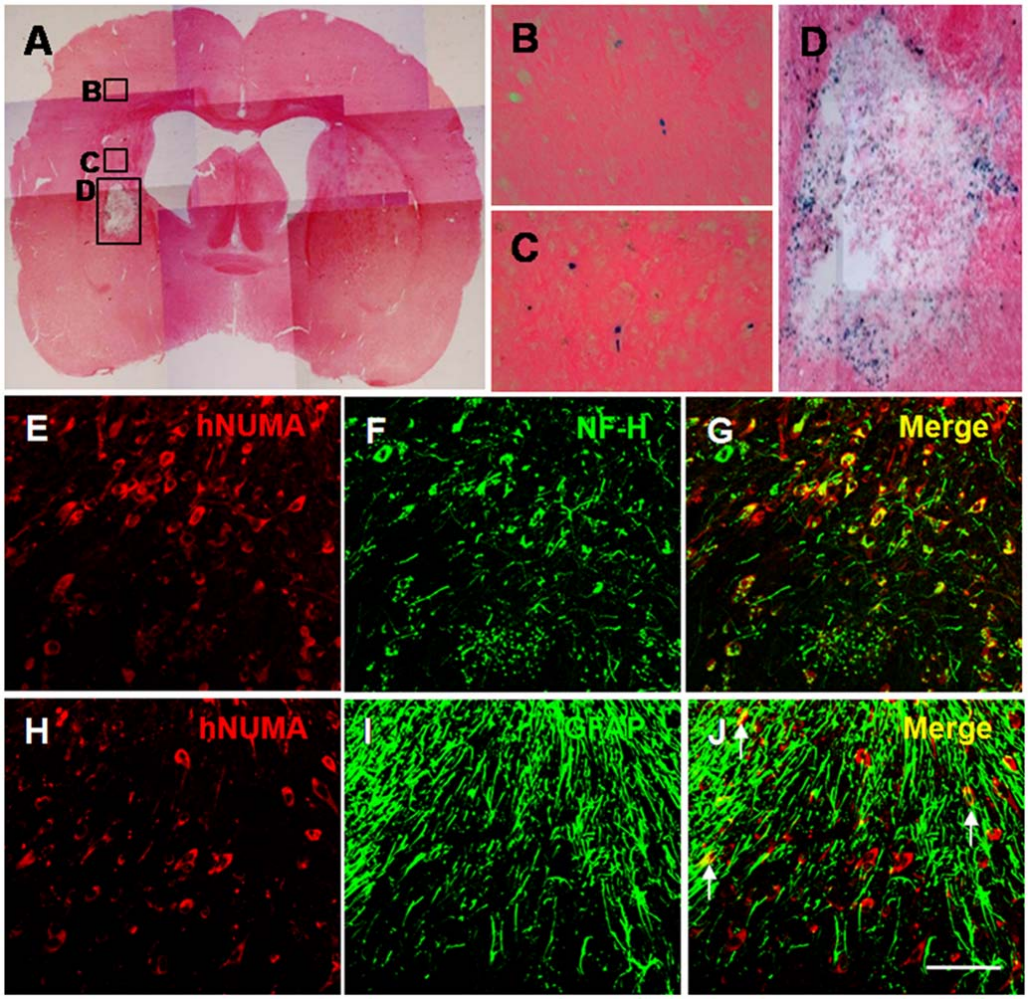
LacZ (beta-galactosidase)-labeled F3.Akt1 human NSCs in intracerebral hemorrhage (ICH) mouse brain at 2 weeks post-transplantation. A: One week after an ICH lesion (an intrastriatal injection of collagenase), LacZ -labeled F3.Akt1 NSCs were transplanted into the cortex overlying the ICH lesion. Two weeks post-transplantation, LacZ-positive F3.Akt1 NSCs were found to migrate extensively into the hemorrhage core and surrounding brain sites. Bar indicates 50 µm. B–D: Higher magnification of indicated areas. Bar indicates 20 µm. E–G: Double immunofluorescent staining of engrafted F3.Akt1 human NSCs in ICH mouse brain 8 weeks post-transplantation. F3.Akt1 NSCs in the lesion sites are found to differentiate into neurons as shown by double staining of human nuclear matrix antigen (hNuMA) and neurofilament protein (NF-H, a neuron specific marker). H–J: F3.Akt1 human NSCs in the lesion sites are found to differentiate into astrocytes (arrows) as shown by double staining of human nuclear matrix antigen (hNuMA) and glial fibrillary acidic protein (GFAP, an astrocyte specific marker). Bar indicates 20 µm.

At 7 days after induction of experimental ICH, 2×10^5^ cells/2 µl of F3 or F3.Akt1 hNSCs were transplanted into ICH mouse cerebral cortex overlying hemorrhage lesion site, 2 mm cranial to the hemorrhagic lesion. LacZ+ human NSCs migrated selectively to the hemorrhagic core and also located on the border of the hemorrhagic core and further away from the injection sites (Figures 4A–C). A large number of transplanted hNuMA (human specific nuclear matrix antigen)-positive F3.Akt1 cells (35–45%) differentiated into NF-H+ neurons in the peri-hematomal sites (Figures 4D–F). While only a small number of transplanted hNuMA+ F3.Akt1 cells (∼4%) were GFAP+ astrocytes and the hNuMA+/GFAP+ double-positive cells were found along the border of hemorrhagic core (Figures 4G–I). These results indicate that a large portion of grafted F3.Akt1 cells differentiate into either neurons or astrocytes in response to signals from the local microenvironment provided by the hemorrhagic lesion.

### Survival of transplanted F3 and F3.Akt1 hNSCs in ICH brain

**Figure 5:**
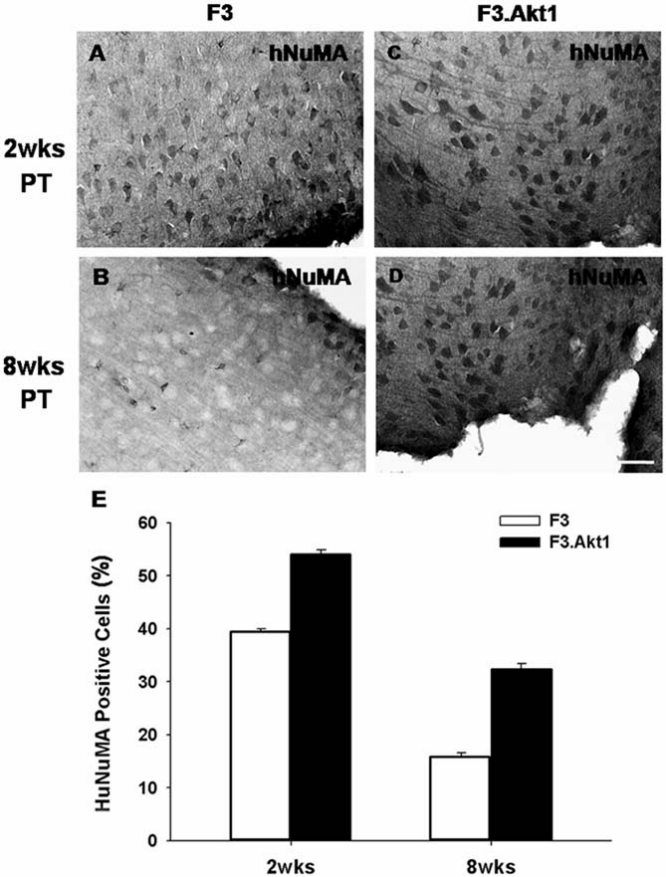
At 2 weeks post-transplantation (PT) in the hemorrhage core border areas, difference in number of human nuclear matrix antigen (hNuMA)-positive cells between ICH-F3 control group (A) and ICH-F3.Akt1 group (C) is not apparent, but at 8 weeks PT number of hNuMA-positive cells is much higher in F3.Akt1-ICH group (D) as compared to control F3-ICH group (B). Bar indicates 100 µm. E: Percentage of hNuMA-positive cells found in hemorrhage core border area is higher in ICH-F3.Akt1 group as compared to ICH-F3 group at both 2 and 8 weeks post-transplantation.

**Figure 6:**
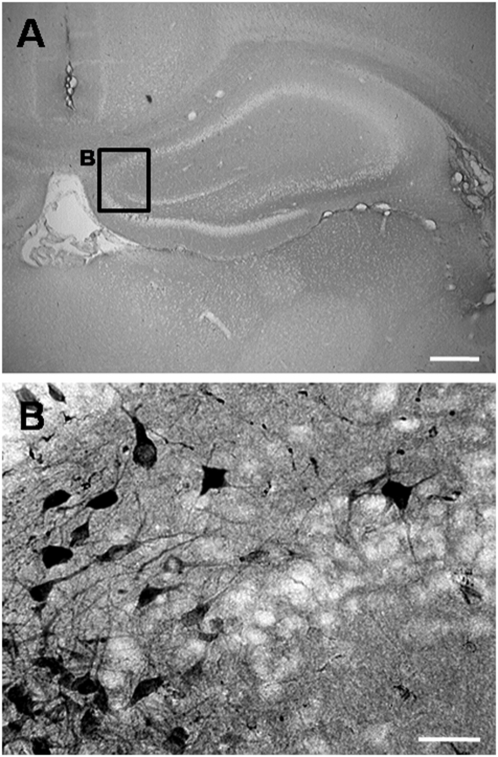
Survival of grafted F3.Akt1 human NSCs in hippocampus was demonstrated by immunoperoxidase microscopy of hNuMA at 8 weeks post-transplantation (PT). A: Lower magnification of hippocampus 8 weeks PT. Bar indicates 1 mm. B: F3.Akt1 human NSCs grafted in cortex overlying striatum were found to migrate extensively to hippocampus 8 weeks PT. Bar indicates 50 µm.

Identification of grafted F3.Akt1 hNSCs in ICH brain was determined by immunostaining with hNuMA (Figure 5). Total numbers of hNuMA-positive F3 and F3.Akt1 hNSCs in the ICH brain sections were carried out using stereological estimation at 2- and 8-weeks post-transplantation (PT). Cell survival rate of F3.Akt1 hNSCs at 2-weeks PT is 107,770±2040 cells (54% of the initial population of 200,000 cells) and at 8 weeks PT the number is 64,890±1940 cells (33% of the initial population), while in control parental F3 hNSCs cell survival at 2 weeks PT is 78,320±1250 cells (39% of the initial population of 200,000 cells) and 32,540±4920 cells (16% of the initial population of 200,000 cells) at 8-weeks (p<0.05) (Figure 5). These results indicate that Akt1 overexpression in hNSCs resulted in a 40% increase in cell survival of transplanted hNSCs at 2 weeks PT and 100% increase at 8 weeks PT. In addition, F3.Akt1 human NSCs grafted in cortex overlying striatum were found to migrate extensively to hippocampus 8 weeks PT (Figure 6) indicating that F3.Akt1 hNSCs are capable of migrating to distant anatomical site.

To determine whether Akt1 expression in F3.Akt1 cells induces their own proliferation, expression of cell proliferation marker Ki-67 was examined in ICH brain sections (Figure S1). Transplanted F3.Akt1 cells identified as hNuMA-positive cells were immunoreaction-negative for proliferation marker Ki-67 indicating that the grafted F3.Akt1 cells did not continue to proliferate following transplantation. Furthermore there was no sign of tissue distortion or tumor formation in brain of ICH animals grafted with F3 or F3.Akt1 hNSCs 6 months PT (Figure S2). Good survival of hNSCs was found in the hNSC injection path into striatum two days after transplantation (Figure S3).

## Discussion

In the present study, mouse ICH model was used to provide proof-of-principle that hNSCs genetically modified to express Akt1, a general mediator of cell survival signals, can be transplanted in the brain of animal models of neurological diseases, and produces beneficial effects of increased survival of grafted NSCs and consequent functional recovery. We demonstrate that brain transplantation of hNSCs overexpressing Akt1 in the collagenase-induced ICH mice resulted in improvement in motor performance as determined by rotarod and limb placement tests, increased survival of grafted NSCs, and differentiation of grafted NSCs into neurons and astrocytes. These results are consistent with previous studies that Akt1, protein which is known as a general mediator of cell survival signals, promotes the survival of CNS neurons *in vitro* [23], [27]–[29], and provides favorable clinical outcome in animal models of ischemia [36].

In the present study we exposed F3 and F3.Akt1 hNSCs to hydrogen peroxide injury *in vitro* to study the neuroprotectiion provided by Akt1 under the conditions of oxidative stress, and the results indicate that F3.Akt1 cells showed a higher survival rate following H_2_O_2_ -induced injury as compared to the F3 controls (Figs. 2A–C). Following H_2_O_2_ treatment, phosphorylation of Akt1 and concomitant inactivation of caspase-3 were found in F3.AKt1 cells, while in control F3 cells non-phosphorylation of Akt1 and an increase in active fragment of caspase 3 were found (Fig. 2D). It is known that Akt1 phosphorylates caspase-9 at Ser-196, thereby blocking cytochrome c-mediated caspas-9 activation and inactivation of caspase-3 leading to inhibition of proapoptotic signals [37]. Recent studies have also shown that antioxidant enzymes such as glutathione peroxidase-1 (Gpx1), Cu/Zn-superoxide dismutase (Cu/Zn-SOD) and heme oxygenase-1 (HO-1) are target substrates of activated Akt and results in modulation of redox system and reduced toxic levels of reactive oxygen species (ROS) in various cell types [38]–[40].

From as early as 8-day post-transplantation (PT) to 8-weeks PT, F3.Akt1 hNSCs induced an increased survival of transplanted NSCs in the host brain. Survival of transplanted F3.Akt1 hNSCs in ICH mouse brain was identified by human nuclear matrix antigen (hNuMA)- or LacZ/β-gal-positive reaction. The cell count in ICH brain indicates that 40% increase in cell survival in F3.Akt1 group over control F3 group and 100% increase at 8-weeks PT. It should be noted that the majority of grafted F3.Akt1 cells differentiated into either neurons (β-tubulin III-, NF-L- or NF-H-positive) or astrocytes (GFAP-positive) along the border of lesion sites (Fig. 4). In addition, a majority of grafted F3.Akt1 hNSCs differentiated into either neurons or astrocytes in response to signals provided in the local microenvironment.

Previous studies have reported that the activation of PI3K/Akt signaling axis promotes growth and survival of tumor cells, and genetic perturbation of this pathway increases the survival of cancer cells [41]–[43]. For that reason, we were very concerned about possible tumorigenesis in the animals transplanted with F3.Akt1 hNSCs. Transplanted F3 or F3.Akt1 hNSCs in ICH lesion sites were immunoreaction-negative for cell proliferation marker Ki-67 (supplementary Fig. 1) indicating that hNSCs do not proliferate actively in vivo environment. In addition, none of the animals grafted with F3.Akt1 or F3 hNSCs showed tumor formation upon histological examination even in animals with 6-months PT (Supplementery Fig. 2).

Akt activity is induced following PI3K activation in various growth factor-mediated signaling cascades [23]. The PI3K-Akt signal pathway is well-known for the cell survival and it exhibits anti-apoptotic effects against a variety of apoptotic paradigms including withdrawal of extracellular signaling factors, oxidative and osmotic stress, irradiation and ischemic shock [24]–[27]. Akt regulates cell growth and survival mechanisms via phosphorylation of a large number of substrates such as Forkhead transcription factors (FOXO), GSK-3, BAD, caspase-9 and MDM2, and many of these proteins contribute to antiapoptotic signaling in various cell types [24]–[27]. Activation of PI3K/Akt signaling pathway promotes extended survival of neuronal cell types from cell death caused by injuries, including cerebellar granule cells [23] and hippocampal neurons [27]–[29].

Akt1 as well as its downstream molecules offer great promise for the development of therapeutics against a number of neurological disorders such as stroke, spinal cord injury and neurodegenerative diseases such as Alzheimer disease, Parkinson disease and ALS. Initially believed to have cellular functions directed primarily toward cell survival and growth, Akt1 is now seen as a potential broad cytoprotective agent. Akt1 can offer cellular protection not only through the modulation of intrinsic apoptotic machinery, but also through the activation of survival signal pathways. Akt1 drives cellular survival signals through a series of distinct pathways that involve the Forkhead family of transcription factors, GSK-3β, β-catenin, eIF2B, c-Jun, CREB, Bad, IKK, p53, and JIPs [25], [26]. Yet, it is evident that further work that clarifies the cellular environment controlled by Akt1 will be of exceptional value to refine our knowledge of Akt1 and to maximize the potential of this protein as a therapeutic agent.

## Supporting Information

Figure S1 Whether Akt1 expression in F3 or F3.Akt1 NSCs causes proliferation in vivo, cell proliferation marker Ki-67 was examined immunochemically in brain sections. Transplanted F3 or F3.Akt1 cells (hNuMA-positive/green) are immunoreaction-negative for cell proliferation marker Ki-67 (red). Ki-67-positive cells represent host mouse brain cells. A: Control parental F3 NSCs. B: F3/Akt1 NSCs.
(0.30 MB TIF)

Figure S2 A: Hematoxylin and Eosin-stained section of mouse brain transplanted with F3.Akt1 NSCs. Six months post-transplantation. Transplantation of v-myc-immortalized F3 or F3.Akt1 human NSCs did not cause tumor formation in the brain. B: Inset indicates higher magnification of the marked area.
(1.21 MB TIF)

Figure S3 A: A schematic drawing of the ICH brain in which β-gal-labeled human NSCs were transplanted. Two days post-transplantation. The circle in the neostriatum represents hemorrhagic core, and the marked areas B and C represent NSC injection path. B–C: Higher magnification of the marked areas where large number of β-gal-labeled NSCs is found.
(0.42 MB TIF)
