# Supplementary figures and images for: Human Neural Stem Cells Genetically Modified to Overexpress Akt1 Provide Neuroprotection and Functional Improvement in Mouse Stroke Model
Source: PLoS One. 2009 May 18;4(5):e5586. doi: 10.1371/journal.pone.0005586 (PMC2679145; doi:10.1371/journal.pone.0005586)

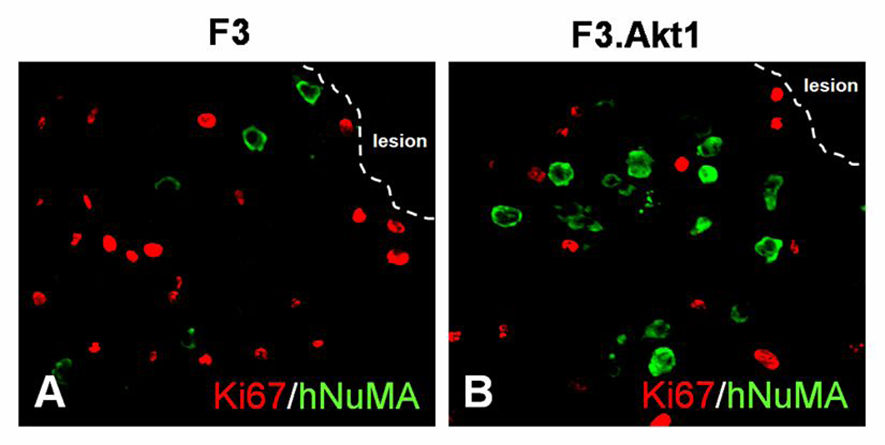

Supplement: Figure S1 — Whether Akt1 expression in F3 or F3.Akt1 NSCs causes proliferation in vivo, cell proliferation marker Ki-67 was examined immunochemically in brain sections. Transplanted F3 or F3.Akt1 cells (hNuMA-positive/green) are immunoreaction-negative for cell proliferation marker Ki-67 (red). Ki-67-positive cells represent host mouse brain cells. A: Control parental F3 NSCs. B: F3/Akt1 NSCs. (0.30 MB TIF) [file pone.0005586.s001.tif]

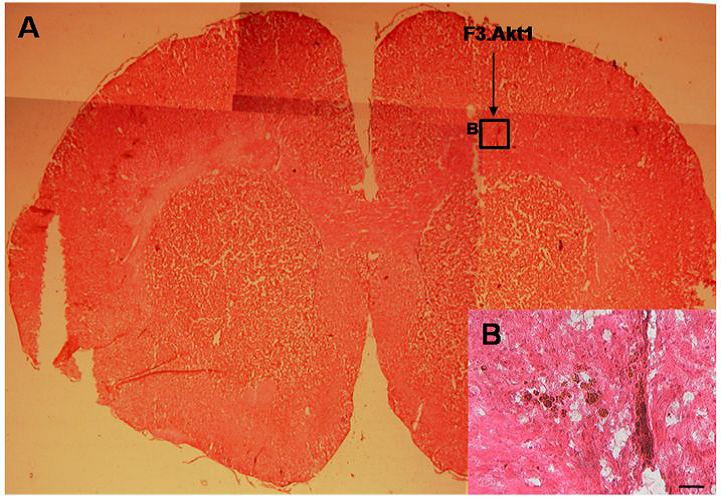

Supplement: Figure S2 — A: Hematoxylin and Eosin-stained section of mouse brain transplanted with F3.Akt1 NSCs. Six months post-transplantation. Transplantation of v-myc-immortalized F3 or F3.Akt1 human NSCs did not cause tumor formation in the brain. B: Inset indicates higher magnification of the marked area. (1.21 MB TIF) [file pone.0005586.s002.tif]

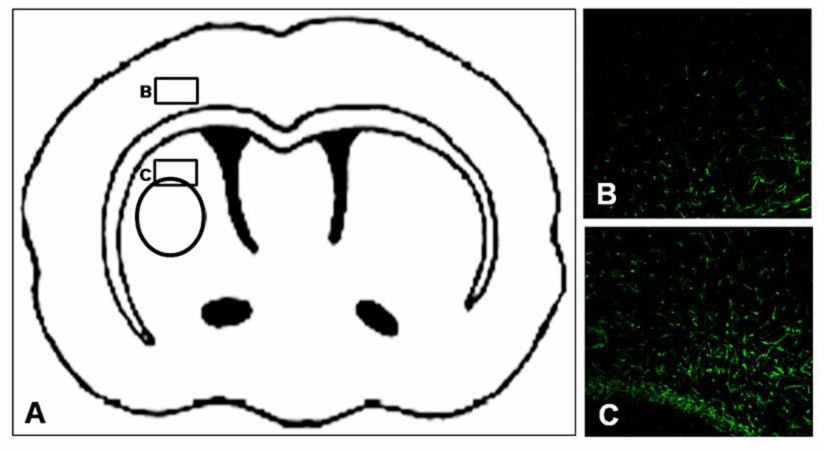

Supplement: Figure S3 — A: A schematic drawing of the ICH brain in which β-gal-labeled human NSCs were transplanted. Two days post-transplantation. The circle in the neostriatum represents hemorrhagic core, and the marked areas B and C represent NSC injection path. B–C: Higher magnification of the marked areas where large number of β-gal-labeled NSCs is found. (0.42 MB TIF) [file pone.0005586.s003.tif]
